# O.2.3-1 Stand Up for Health: process evaluation of an intervention to reduce sedentary behaviour in contact centres

**DOI:** 10.1093/eurpub/ckad133.127

**Published:** 2023-09-11

**Authors:** Divya Sivaramakrishnan, Jillian Manner, Graham Baker, Richard Parker, Scott Lloyd, Ruth Jepson

**Affiliations:** Scottish Collaboration for Public Health Research and Policy, University of Edinburgh, UK; Scottish Collaboration for Public Health Research and Policy, University of Edinburgh, UK; Physical Activity for Health Research Centre, University of Edinburgh, UK; Edinburgh Clinical Trials Unit, Usher Institute, University of Edinburgh, UK; Public Health South Tees, Middlesbrough Council and Redcar & Cleveland Borough Council, Middlesbrough, UK; divya.sivaramakrishnan@ed.ac.uk; Scottish Collaboration for Public Health Research and Policy, University of Edinburgh, UK

## Abstract

**Purpose:**

Contact centres have been identified as high-pressured workplaces where staff are sedentary, and one in four experience musculoskeletal problems. Stand Up for Health (SUH) is an intervention developed using the 6SQuID intervention development framework to target sedentary behaviour in contact centres. It is an adaptive intervention based on the Social Cognitive Theory and the Social Ecological Model. The aim of this study was to test the acceptability and feasibility of implementing SUH in contact centres.

**Methods:**

The study used a stepped-wedge cluster randomised trial design and included eleven UK-based contact centres. Intervention implementation involved working with contact centre stakeholders to develop a customised action plan that aligned with SUH’s theory of change. This was operationalised to include two workshops, creating a SUH committee, and loaning equipment (desk-risers, exercise equipment) to each centre. During the pandemic, online staff consults replaced these activities. The process evaluation adopted the RE-AIM framework to understand acceptability and feasibility of implementing the SUH intervention. Interviews and focus groups were conducted with 33 staff members and stakeholders, and 96 participants completed an activity preference questionnaire. Qualitative data were analysed using a codebook thematic analysis approach and descriptive statistics were used to describe activity preferences.

**Results:**

The intervention was acceptable and feasible to deliver, and most contact centres implemented several activities aligned with each level of the programme’s theory of change. All centres reported that more than 50% of staff participated in at least one SUH activity (during the pre-lockdown period). Perceived benefits such as reduced sedentary behaviour, increased physical activity, and improved staff morale and mood were reported by contact centre staff and stakeholders. Participants felt that SUH was particularly significant because it increased awareness of sedentary behaviour, encouraged movement, and helped staff manage stress.

**Conclusions:**

SUH is an adaptive multicomponent programme that considers the culture and contexts of contact centres. The programme shows potential as an appealing and acceptable intervention, impacting several wellbeing outcomes. Next steps include adapting the intervention for the post-pandemic work context, and further pilot testing before large scale evaluation.

**Funding Source:**

National Institute for Health Research (NIHR) [PHR project grant: 17/149/19]

